# Gastric Non-Helicobacter pylori Urease-Positive Staphylococcus epidermidis and Streptococcus salivarius Isolated from Humans Have Contrasting Effects on H. pylori-Associated Gastric Pathology and Host Immune Responses in a Murine Model of Gastric Cancer

**DOI:** 10.1128/msphere.00772-21

**Published:** 2022-02-09

**Authors:** Zeli Shen, JoAnn Dzink-Fox, Yan Feng, Sureshkumar Muthupalani, Anthony J. Mannion, Alexander Sheh, Mark T. Whary, Hilda R. Holcombe, Blanca M. Piazuelo, Luis E. Bravo, Christine Josenhans, Sebastian Suerbaum, Keith T. Wilson, Richard M. Peek, Timothy C. Wang, James G. Fox

**Affiliations:** a Division of Comparative Medicine, Massachusetts Institute of Technology, Cambridge, Massachusetts, USA; b Division of Gastroenterology, Vanderbilt University Medical Center, Nashville, Tennessee, USA; c Department of Pathology, Universidad del Valle, Cali, Colombia; d Medical Microbiology and Hospital Epidemiology, Max von Pettenkofer-Institute, Ludwig Maximilians University, Munich, Germany; e Division of Gastroenterology and Irvine Cancer Research Center, Columbia University, New York, New York, USA; University of Michigan—Ann Arbor

**Keywords:** *H. pylori*, microbiome, *S. epidermidis*, *S. salivarius*, INS-GAS mice

## Abstract

In populations with similar prevalence of Helicobacter pylori infection, cancer risk can vary dramatically. Changes in composition or structure of bacterial communities in the stomach, either at the time of exposure or over the course of H. pylori infection, may contribute to gastric pathology. In this study, a population of 37 patients from the low-gastric-cancer-risk (LGCR) region of Tumaco, Colombia, and the high-gastric-cancer-risk (HGCR) region of Túquerres, Colombia, were recruited for gastric endoscopy. Antral biopsy specimens were processed for histology and bacterial isolation. Fifty-nine distinct species among 26 genera were isolated by aerobic, anaerobic, and microaerobic culture and confirmed by 16S rRNA analysis. Urease-positive Staphylococcus epidermidis and Streptococcus salivarius were frequently isolated from gastric biopsy specimens. We asked whether coinfection of H. pylori with urease-positive *S. salivarius* and/or S. epidermidis had a demonstrable effect on H. pylori*-*induced gastritis in the germfree (GF) INS-GAS mouse model. Coinfections with *S. salivarius* and/or S. epidermidis did not affect gastric H. pylori colonization. At 5 months postinfection, GF INS-GAS mice coinfected with H. pylori and *S. salivarius* had statistically higher pathological scores in the stomachs than mice infected with H. pylori only or H. pylori with S. epidermidis (*P* < 0.05). S. epidermidis coinfection with H. pylori did not significantly change stomach pathology, but levels of the proinflammatory cytokine genes *Il-1β*, *Il-17A *, and *Il-22* were significantly lower than in H. pylori-monoinfected mice. This study demonstrates that non-H. pylori urease-positive bacteria may play a role in the severity of H. pylori*-*induced gastric cancer in humans.

**IMPORTANCE** Chronic infection with H. pylori is the main cause of gastric cancer, which is a global health problem. In two Colombian populations with high levels of H. pylori prevalence, the regional gastric cancer rates are considerably different. Host genetic background, H. pylori biotype, environmental toxins, and dietary choices are among the known risk factors for stomach cancer. The potential role of non-H. pylori gastric microbiota in gastric carcinogenesis is being increasingly recognized. In this study, we isolated 59 bacterial species from 37 stomach biopsy samples of Colombian patients from both low-gastric-cancer-risk and high-gastric-cancer-risk regions. Urease-positive S. epidermidis and *S. salivarius* commonly cultured from the stomachs, along with H. pylori, were inoculated into germfree INS-GAS mice. *S. salivarius* coinfection with H. pylori induced significantly higher gastric pathology than in H. pylori-monoinfected mice, whereas S. epidermidis coinfection caused significantly lower H. pylori-induced proinflammatory cytokine responses than in H. pylori-monoinfected mice. This study reinforces the argument that the non-H. pylori stomach microflora play a role in the severity of H. pylori-induced gastric cancer.

## INTRODUCTION

Recent studies in humans indicate that gastric colonization by non-Helicobacter pylori bacteria, many of which normally colonize the lower bowel and oral cavity, could modulate the risk for gastric adenocarcinoma (GAC) (1, 2). Dyspeptic H. pylori patients treated with acid-suppressive drugs have significant increases in non-H. pylori bacteria colonizing the stomach and higher levels of inflammatory cytokines, which confer a greater risk of atrophic gastritis. These data suggest that non-H. pylori bacteria colonize the stomach and promote H. pylori gastric carcinogenesis (3–5).

Inhabitants of Túquerres in the Colombian Andes have a 25-fold higher risk of gastric cancer than inhabitants of the coastal town of Tumaco, despite a similar prevalence of H. pylori infection (6). We recently published a paper describing the gastric microbiome in H. pylori*-*infected individuals living in the low- and high-risk areas of Colombia (7). Twenty individuals from each town, matched for age and sex, were selected, and gastric microbiota analyses were performed by deep sequencing of amplified 16S rRNA. In parallel, analyses of H. pylori status, detection of carriage of the *cag* pathogenicity island, and assignment of H. pylori to phylogeographic groups were performed to test for correlations between H. pylori strain properties and microbiota composition (7). Multiple operational taxonomic units (OTUs) were detected exclusively in either Tumaco or Túquerres. Two OTUs, Leptotrichia wadei and *Veillonella* spp., were significantly more abundant in Túquerres, and 16 OTUs, including a Staphylococcus species closely related to Staphylococcus epidermidis, were significantly more abundant in Tumaco. Four members of the genus Streptococcus were correlated with multifocal atrophic gastritis with intestinal metaplasia (MAG-IM). There was no significant correlation between H. pylori phylogeographic population or carriage of the *cag* pathogenicity island (PAI) and microbiota composition (7).

Studies have demonstrated that selected bacterial species have a direct effect on growth inhibition of H. pylori (8). In particular, two strains of S. epidermidis inhibited growth of all H. pylori strains (27 strains in one assay and 35 strains in another assay) (8). Streptococcus bovis*/*Streptococcus gallolyticus has been associated with malignant gastrointestinal diseases, especially colon cancer, and has been detected in the stomachs of patients with gastric cancer (5). These findings suggested the importance of non-H. pylori bacteria in patients with H. pylori-associated gastric diseases. In this study, we used culture-dependent methods to isolate non-H. pylori bacteria from gastric biopsy samples collected from high-gastric-cancer-risk (HGCR) and low-gastric-cancer-risk (LGCR) patients with or without H. pylori infection. Because urease-positive bacteria, like H. pylori, have a selective advantage in colonizing the acidic milieu of the stomach, we tested these bacterial isolates for urease activity. Two urease-positive, Gram-positive organisms, Streptococcus salivarius (isolated from high-risk patients) and Staphylococcus epidermidis (commonly isolated from LGCR patients), were inoculated into germfree (GF) INS-GAS mice infected with H. pylori to ascertain whether these bacteria could influence H. pylori*-*associated pathogenesis.

## RESULTS

### Bacterial species identification in gastric antral biopsy specimens.

No bacterial growth was detected in two of the 37 biopsy samples using three different culture conditions. Of the remaining 35 biopsy specimens, 59 distinct species from 26 genera and 4 phyla were identified by culture and confirmed by 16S rRNA sequence analysis. Seven genera in *Actinobacteria*, one genus in *Bacteroidetes*, 11 genera in *Firmicutes*, and seven genera in *Proteobacteria* were identified (Table 1). The prevalence of bacterial species isolated from the biopsy samples from the HGCR and LGCR populations is presented in Table S1 in the supplemental material.

**TABLE 1:** Bacterial genera isolated from biopsy samples

Genus	Total no.^*a*^	HGCR		LGCR	
		HP^+^ (*n*/10)	HP^−^ (*n*/9)	HP^+^ (*n*/10)	HP^−^ (*n*/8)
*Actinomyces*	4	4			
*Aerococcus*	1		1		
*Arthrobacter*	1	1			
*Atopobium*	3	2	1		
*Bacillus*	18	3	4	6	5
Campylobacter	1	1			
*Citrobacter*	1		1		
*Enterococcus*	1				1
Escherichia	1		1		
*Gemella*	1	1			
*Gordonia*	2		1	1	
*Helicobacter*	17	7		10	
*Lactobacillus*	2	1			1
*Mogibacterium*	1	1			
*Moraxella*	1	1			
*Neisseria*	2	1		1	
*Paenibacillus*	1		1		
*Prevotella*	2	1		1	
*Propionibacterium*	2		1	1	
*Rothia*	2	2			
*Solobacterium*	1	1			
Staphylococcus	14	2	2	4	6
Streptococcus	17	6	5	2	4
*Streptomyces*	2	2			
*Veillonella*	7	4		1	2
*Xanthomonas*	1		1		

a Number of biopsy specimens with the indicated bacterial genus. *n*, number of patients; HP^+^, *H. pylori*-positive patients; HP^−^, *H. pylori*-negative patients.

10.1128/mSphere.00772-21.1 TABLE S1 Prevalence of bacteria isolated from stomach biopsy samples of H. pylori-positive and H. pylori-negative patients in HGCR and LGCR populations. n*, Number of biopsy specimens with a distinct bacterial genus or species. Download Table S1, DOCX file, 0.02 MB. Copyright © 2022 Shen et al. 2022 Shen et al. https://creativecommons.org/licenses/by/4.0/ This content is distributed under the terms of the Creative Commons Attribution 4.0 International license.

**FIG 1:**
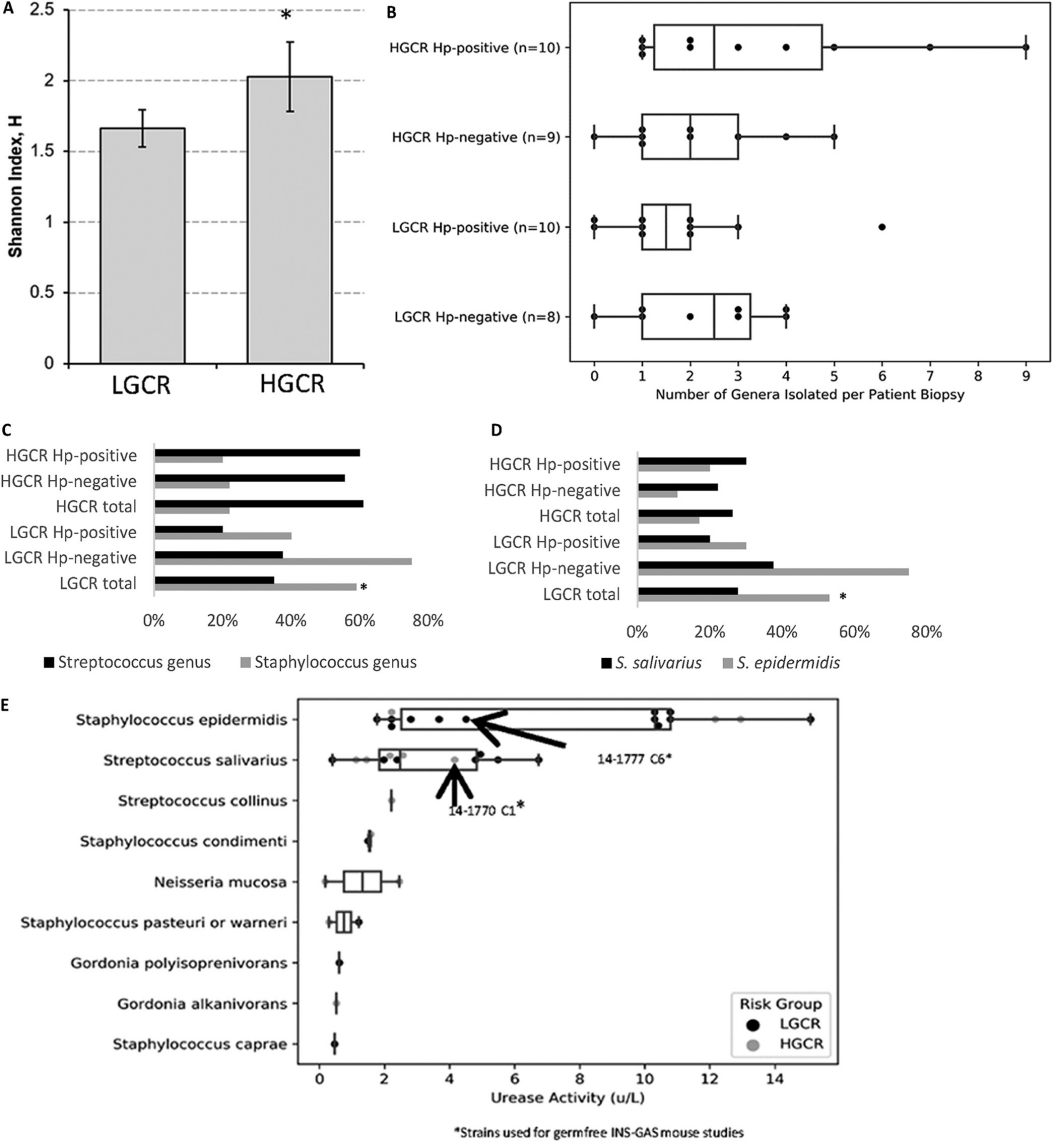
(A) Shannon diversity index for the LGCR and HGCR samples. *, *P* < 0.0001 (implies that HGCR samples have more diversity in bacterial richness and evenness). (B) Non-H. pylori bacterial genera isolated from biopsy samples. (C) Prevalence of Streptococcus and Staphylococcus in LGCR and HGCR populations. Staphylococcus was significantly higher in LGCR patients. *, *P* < 0.05. (D) Prevalence of *S. salivarius* and S. epidermidis in LGCR and HGCR populations. *, *P* < 0.05. (E) Urease activities of non-H. pylori isolates cultured from gastric biopsy specimens. H. pylori strain SS1 was used as a positive control and had a urease activity of 262 U/L; Escherichia coli ATCC 11775 was used as a negative control. *, strain used in germfree INS-GAS mouse studies.

The isolated bacterial species from LGCR and HGCR regions were also used to determine differences in diversity using community ecology metrics. The results suggested that the LGCR and HGCR regions had statistically different ecological communities based on culture results. The Shannon diversity indexes for HGCR and LGCR regions were 2.027 and 1.664, respectively, indicating an increased diversity in HGCR (Fig. 1A). Dividing the bacteria into subsets based on region and the patient’s H. pylori status, H. pylori infection increased the diversity of culturable organisms compared to that in uninfected individuals from the same geographic location. This finding can potentially signify that H. pylori infection can modify the types of bacteria that are cultured using standard clinical microbiological techniques.

For individual biopsy samples, 0 to 9 different bacterial genera were identified. The median number of genera identified was 2, and 85% of samples had fewer than 5 bacterial genera identified. One sample from an H. pylori-positive patient from the HGCR group had 9 bacteria genera identified. There was no significant difference in the numbers of bacterial genera isolated from different regions (Fig. 1B). The five most commonly isolated non-H. pylori genera from patients were *Bacillus* (*n* = 18), Streptococcus (*n* = 17), Staphylococcus (*n* = 14), *Veillonella* (*n* = 7), and *Actinomyces* (*n* = 4). The prevalence of several bacterial genera in LGCR was higher. *Bacillus* spp. were isolated from 65% of LGCR biopsy samples but from only 39% of HGCR biopsy samples. Among *Bacillus* spp., Bacillus pumilus was isolated from 41% of the biopsy samples in the LGCR region but only 5% of the biopsy samples in the HGCR region. *Staphylococci* spp. were isolated from 59% of LGCR biopsy samples and 22% of HGCR samples. S. epidermidis was isolated from 53% of the LGCR biopsy samples but only 17% of the HGCR biopsy samples. Streptococcus spp. were isolated from 61% of biopsy samples from HGCR individuals and only 35% of samples from LGCR individuals. Both Staphylococcus spp. and Streptococcus spp. were present in 7 biopsy specimens. (Fig. 1C and D). We used Fisher’s exact test to determine if it was more likely to isolate each of these species from patients in the LGCR or HGCR region. A statistically significant increase in Staphylococcus-positive LGCR samples was observed compared to HGCR samples (*P* < 0.05), and the prevalence of S. epidermidis-positive samples was significantly higher in LGCR than HGCR samples (*P* < 0.05).

### Urease-positive gastric bacteria.

Of the 20 patients who were considered H. pylori positive by histology, H. pylori was isolated from all 10 patients in the LGCR region and 7/10 patients in the HGCR region. Two patients had H. pylori growth only, six had H. pylori plus 1 additional bacterial species, and the remaining 12 had 2 to 9 non-H. pylori species, including 1 to 3 urease-positive species.

Of the 17 H. pylori-negative biopsy specimens, 10 had 1 to 2 urease-positive non-H. pylori species. Urease-positive bacteria commonly isolated included Staphylococcus spp. (Staphylococcus condimenti, S. epidermidis, S. caprae, and S. warneri) and Streptococcus spp. (Streptococcus salivarius and S. mitis), with fewer urease-positive *Gordonia* spp., *Neisseria* spp., Citrobacter freundii, and *Bacillus* spp.

Figure 1E depicts the urease-positive bacterial strains recovered from the biopsy specimens and their average urease activity. H. pylori SS1, used as the positive control, had the strongest urease activity (262 U/L). The urease activities of the Staphylococcus spp. and the Streptococcus spp. were markedly less than that of H. pylori (ranges, 0.46 to 15.1 U/L and 0.4 to 6.74 U/L, respectively). Given the high prevalence of both *S. salivarius* and S. epidermidis in these biopsy specimens, we elected to address whether these urease-positive bacteria had a demonstrable effect on H. pylori pathogenesis in the INS-GAS mouse model.

### Effects of *S. salivarius* and S. epidermidis on H. pylori pathogenesis in GF INS-GAS mice. (i) Gastric colonization with *S. salivarius* and S. epidermidis does not produce gastritis in GF mice.

GF INS-GAS mice were infected with *S. salivarius* MIT 14-1770 C1 from the HGCR region (S.sal-A) and S. epidermidis MIT 14-1777 C6 from the LGCR region (S.epi-A) to determine whether a mixed population of urease-positive bacteria would induce gastritis in the absence of H. pylori infection. Gastric cultures from mice euthanized at 5 months postinoculation (p.i.) confirmed persistent colonization with both bacteria. Infected mice had pathology scores similar to those of the control GF mice at this time point (data not shown).

### (ii) Cocolonization with *S. salivarius* and S. epidermidis induced similar gastric pathology but decreased inflammatory markers in H. pylori-infected mice.

**FIG 2:**
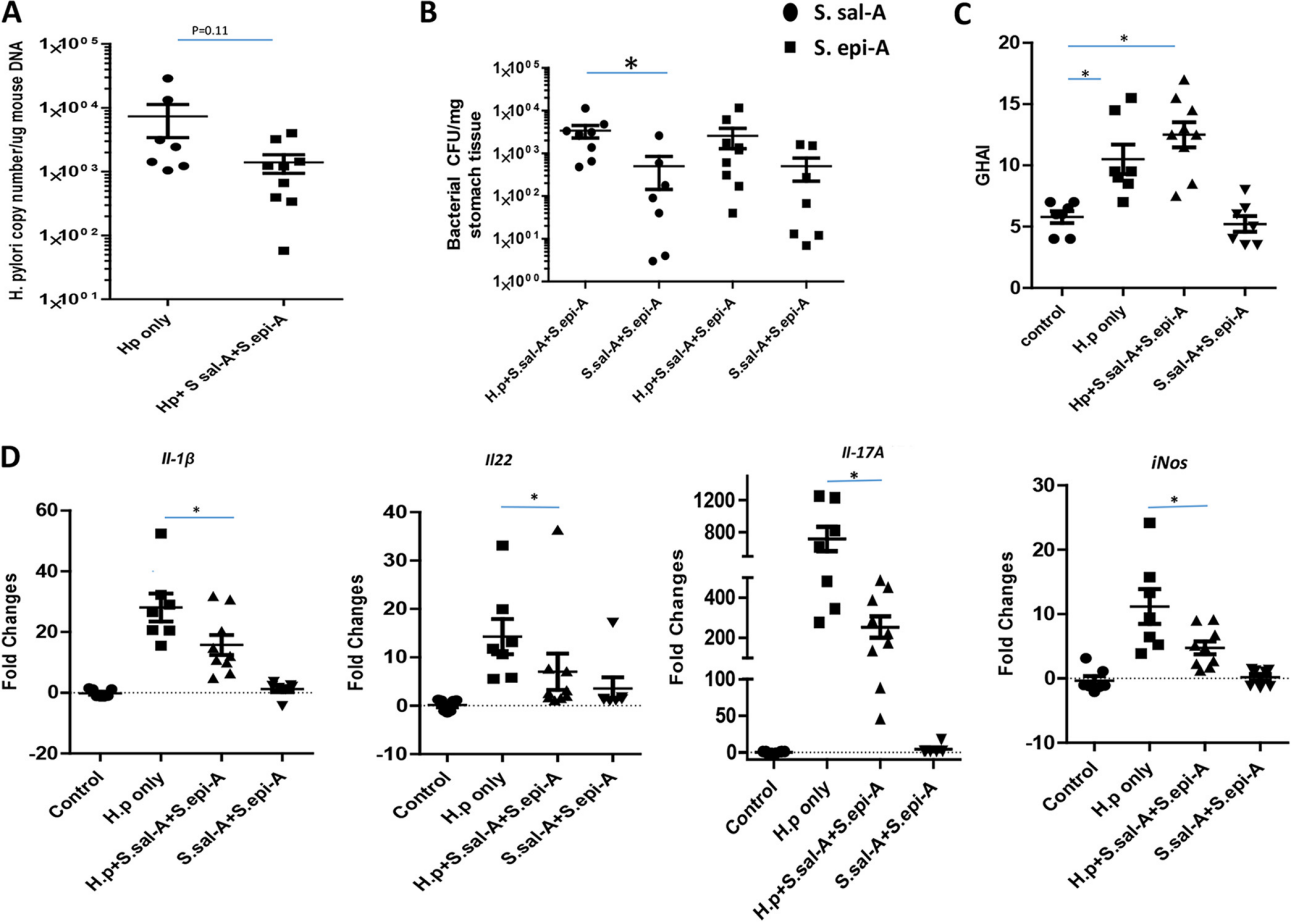
Experiment 1. Each point in the figure indicates an individual mouse. *, *P* < 0.05. (A) Quantitative PCR for H. pylori colonization. Cocolonization of H. pylori-infected mice with *S. salivarius* and S. epidermidis did not significantly affect H. pylori colonization levels. (B) *S. salivarius* (S.sal-A) and S. epidermidis (S.epi-A) persistently colonized the gastrointestinal tracts of GF mice coinfected with H. pylori. *S. salivarius* colonization levels in the stomachs of infected mice were significantly higher in the group coinfected with H. pylori than the *S. salivarius*-S. epidermidis group. (C) H. pylori-monoinfected mice had robust stomach pathology compared to control mice, but the difference was not significant compared to mice coinfected with H. pylori and *S. salivarius* (S.sal-A) and S. epidermidis (S.epi-A). (D) Compared to H. pylori-monoinfected mice, mice coinfected with all three bacteria had statistically lower *Il-1β*, *Il-17A*, and *Il-22* cytokine expression levels as well as lower *iNos* expression levels in the gastric tissues (*P* < 0.05). No changes in mRNA expression levels for *Ifn-γ*, *Tnf-α*, *Il-10*, *Il-11*, *Reg-3γ*, *Reg3-β*, and *Stat-3* were seen (Supplemental Fig. S2).

To further assess the role of coinfection in gastritis and gastric cancer, mice were monoinfected with H. pylori or coinfected with H. pylori, S. epidermidis, and *S. salivarius* (Table 2, experiment 1). Control groups were uninfected or dosed with S. epidermidis and *S. salivarius* without H. pylori (Table 2). Mice dosed with H. pylori remained colonized at 5 months p.i. as indicated by H. pylori culture and PCR. The correlation of H. pylori CFU by culture method and DNA copy number in quantitative PCR (qPCR) assay was performed; the strong correlation (*r* = 0.9927) is presented (Fig. S1). We used the qPCR assay to represent H. pylori colonization levels in our study due to the higher sensitivity compared to the H. pylori culture method. H. pylori levels trended lower but were not significantly different between monoinfected mice and mice coinfected with H. pylori, *S. salivarius*, and S. epidermidis (Fig. 2A). *S. salivarius* and S. epidermidis were isolated from the oral cavities, stomachs, and feces of infected mice (data not shown). The colonization levels of S. epidermidis in the stomachs of S. epidermidis- and *S. salivarius*-infected mice and mice coinfected with H. pylori were not significantly different. In contrast, *S. salivarius* colonization levels were significantly higher in the stomachs of mice coinfected with H. pylori than in S. epidermidis- and *S. salivarius*-infected mice (Fig. 2B).

**TABLE 2:** Germfree INS-GAS mouse experiments

Infection status	No. of mice in expt^*a*^:		
	1	2	3
Controls (uninfected)	7 M	10 M/6 F	6 M/7 F
H. pylori SS1	5 M/2 F	10 M/10 F	12 M/8 F
H. pylori SS1 and *S. salivarius* A and S. epidermidis A	4 M/5 F		
*S. salivarius* A and S. epidermidis A	3 M/4 F		
H. pylori SS1 and *S. salivarius* A		11 M/9 F	
H. pylori SS1 and S. epidermidis A		9 M/12 F	
H. pylori SS1 and S. epidermidis B			10 M/10 F
*S. salivarius* A		5 M/4 F^*b*^	
S. epidermidis B			8 M

a M, male mice; F, female mice.

b Experiment conducted on a different date.

10.1128/mSphere.00772-21.2 FIG S1 Correlation assay between copy number in qPCR and CFU in bacterial culture: Fresh cultures of H. pylori SS1 strain were serially diluted (10×) in Brucella broth. Bacterial suspensions were subject in parallel to DNA extraction for qPCR and microanaerobic culture for bacterial CFU. A strong correlation is presented, with a Pearson correlation coefficient factor (*r*) of 0.9927. Download FIG S1, PDF file, 0.1 MB. Copyright © 2022 Shen et al. 2022 Shen et al. https://creativecommons.org/licenses/by/4.0/ This content is distributed under the terms of the Creative Commons Attribution 4.0 International license.

As expected, H. pylori*-*infected mice developed significant gastric pathology compared to controls. H. pylori-infected mice coinfected with *S. salivarius* and S. epidermidis also developed significant pathology compared to controls, but the pathology did not statistically differ from that of mice monoinfected with H. pylori. H. pylori mice with and without *S. salivarius* and S. epidermidis coinfection had similar gastric lesions, including prominent mucosal and submucosal inflammation, lymphoid aggregates, mucosal atrophy, foveolar and glandular hyperplasia, epithelial defects, pseudopyloric hyperplasia, and moderate to severe dysplasia. Mice dually infected with *S. salivarius* and S. epidermidis mice had pathology scores similar to those of the control GF mice (Fig. 2C).

Interestingly, compared to H. pylori-monoinfected mice, mice coinfected with all three bacteria (i.e., H. pylori, *S. salivarius*, and S. epidermidis) had statistically lower levels of expression of the cytokine genes *Il-1β*, *Il-22*, and *Il-17A*, as well as lower *iNos* expression levels, in gastric tissues (*P* < 0.05) (Fig. 2D). *Tnf-α* and *Ifn-γ* expression levels also trended lower, but gastric expression levels between the two groups for *Il-11*, *Reg-3γ, Reg3-β*, and *Stat-3* were not different (Fig. S2).

10.1128/mSphere.00772-21.3 FIG S2 Gastric mRNA expression levels for *Ifn-γ*, *Tnf-α*, *Il-10*, *Il-11*, *Reg-3γ*, *Reg3-β*, and *Stat-3* in H. pylori-monoinfected mice and mice coinfected with H. pylori, *S. salivarius*, and S. epidermidis had no statistically significant differences. Each point in the figure indicates an individual mouse. Download FIG S2, TIF file, 0.1 MB. Copyright © 2022 Shen et al. 2022 Shen et al. https://creativecommons.org/licenses/by/4.0/ This content is distributed under the terms of the Creative Commons Attribution 4.0 International license.

### (iii) Coinfection with H. pylori and *S. salivarius* induced more severe gastric pathology than monoinfection with H. pylori or coinfection with H. pylori and S. epidermidis.

**FIG 3:**
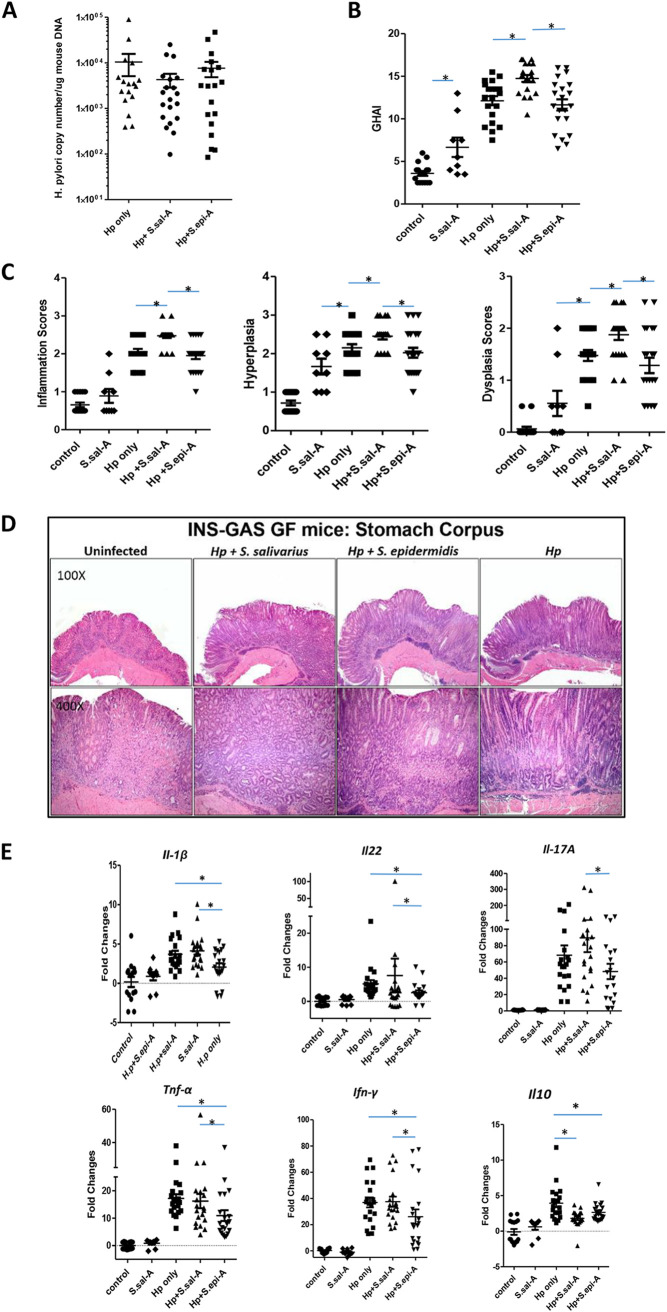
Experiment 2. (A) Quantitative PCR for H. pylori colonization. Cocolonization of H. pylori-infected mice with *S. salivarius* or S. epidermidis did not significantly affect H. pylori colonization levels. (B) Gastric histologic activity index (GHAI) of GF INS-GAS mice. H. pylori-monoinfected mice were not significantly difference from H. pylori-S. epidermidis (S.epi-A)-coinfected mice. H. pylori coinfection with *S. salivarius* (S.sal-A) induced more severe gastric pathology. (C) INS-GAS mice coinfected with H. pylori and *S. salivarius* had significantly higher gastric inflammation, hyperplasia, and dysplasia scores than H. pylori only and H. pylori-S. epidermidis groups. *, *P* < 0.05. (D) H. pylori infection induced moderate gastric pathology compared to control INS-GAS mice. H. pylori coinfection with *S. salivarius* induced more severe pathology than H. pylori only and H. pylori with S. epidermidis. (E) mRNA levels of cytokines in stomachs of INS-GAS mice. Expression levels of the proinflammatory cytokines *Il-1β*, *Il-22*, *Il-17A*, *Ifn-γ*, and *Tnf-α* as well as *Il-10* were statistically lower in the H. pylori-S. epidermidis-coinfected mice than the H. pylori-*S. salivarius*-coinfected mice and H. pylori-monoinfected mice. Each point in the figure indicates an individual mouse. *, *P* < 0.05.

The effects of coinfection with individual bacterial species on H. pylori-induced gastric disease were determined in monoinfected and dually infected mice, as indicated in Table 2, experiments 2 and 3. At 5 months postinfection, H. pylori was successfully recovered from stomach tissue by microaerobic culture (data not shown). There was no statistically significant difference in H. pylori colonization levels in the stomachs by qPCR analysis among the three groups infected with H. pylori (Fig. 3A). Mice monoinfected with *S. salivarius* had increased pathological changes compared with control mice; however, the changes were less severe than those in H. pylori-infected mice (Fig. 3B). All the mice infected with H. pylori had robust pathology compared to uninfected controls at 5 months p.i. Mice infected with H. pylori and *S. salivarius* had statistically higher overall pathology scores than monoinfected H. pylori mice or mice infected with H. pylori and S. epidermidis. Individual indices, including inflammation, hyperplasia, and dysplasia, were higher in H. pylori-*S. salivarius* coinfected mice than monoinfected H. pylori mice or mice coinfected with H. pylori and S. epidermidis (Fig. 3C and D). However, expression levels of tissue cytokines, including those encoded by *Il-1β*, *Il-22*, *Il-17A*, *Tnf-α*, *Ifn-γ*, and *iNos*, were comparable for H. pylori-monoinfected and H. pylori-*S. salivarius*-coinfected mice. Interestingly, H. pylori-*S. salivarius*-coinfected mice had statistically lower expression levels of *Il-10* than H. pylori-monoinfected mice (Fig. 3E).

### (iv) H. pylori and S. epidermidis infected mice have statistically lower overall pathology and cytokine expression levels compared to H. pylori and *S. salivarius* coinfected mice.

The INS-GAS mice coinfected with H. pylori and S. epidermidis had statistically lower pathology scores, including inflammation, hyperplasia, and dysplasia, than mice coinfected with H. pylori and *S. salivarius* (Fig. 3B to D). Similarly, selected proinflammatory tissue cytokine expression levels of *Il-1β*, *Il-22*, *Il-17A*, *Tnf-α*, and *Ifn-γ* were statistically lower in the coinfected H. pylori and S. epidermidis mice compared to coinfected H. pylori and *S. salivarius* mice. Although there was no statistical significance in pathology changes in H. pylori and S. epidermidis coinfected mice compared with monoinfected H. pylori mice, proinflammatory cytokines *Il-1β*, *Il-22*, *Tnf-α*, and *Ifn-γ* were significantly lower in mice coinfected with H. pylori and S. epidermidis (Fig. 3E).

**FIG 4:**
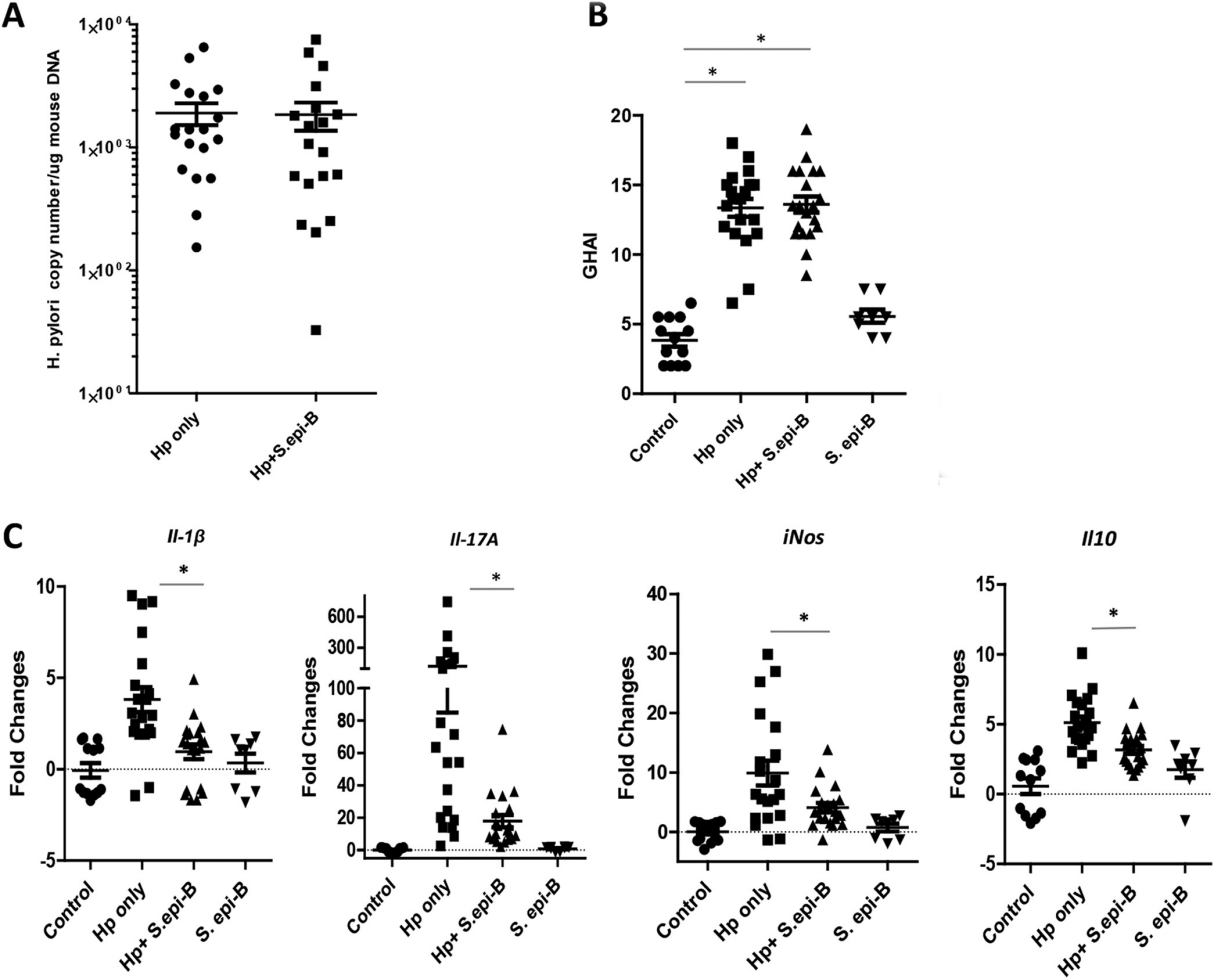
Experiment 3. Each point in the figure indicates an individual mouse. *, *P* < 0.05. (A) Quantitative PCR for H. pylori colonization; (B) gastric histologic activity index (GHAI). (C) Expression levels of proinflammatory cytokine genes *Il-1β* and *Il-17A* as well as *iNos* and Il-10 expression levels in gastric mRNA were lower in H. pylori-S. epidermidis M5-coinfected INS-GAS mice.

In experiment 3 (Table 2), an additional S. epidermidis strain, MIT 14-1779 M5 (LGCR region), was used to investigate if the effects on coinfection with H. pylori were species or strain specific and to investigate the effect of monoinfection with S. epidermidis. Similar to the results in mice infected with S. epidermidis MIT 14-1777 C6, cocolonization of H. pylori-infected mice with S. epidermidis MIT 14-1779 M5 did not significantly affect H. pylori colonization levels (Fig. 4A), No significant differences were observed in gastric pathology in uninfected mice and mice monoinfected with S. epidermidis (Fig. 4B), nor were there significant differences in gastric pathological scores between coinfected mice and H. pylori-monoinfected mice (Fig. 4B). Expression levels of proinflammatory cytokines encoded by *Il-1β* and *Il-17A* as well as those of *iNos* in gastric mRNA were lower in H. pylori-S. epidermidis-coinfected mice (Fig. 4C).

### (v) S. epidermidis reduced proinflammatory anti-H. pylori isotype IgG2a, while *S. salivarius* and S. epidermidis both reduced anti-inflammatory anti-H. pylori isotype IgG1.

**FIG 5:**
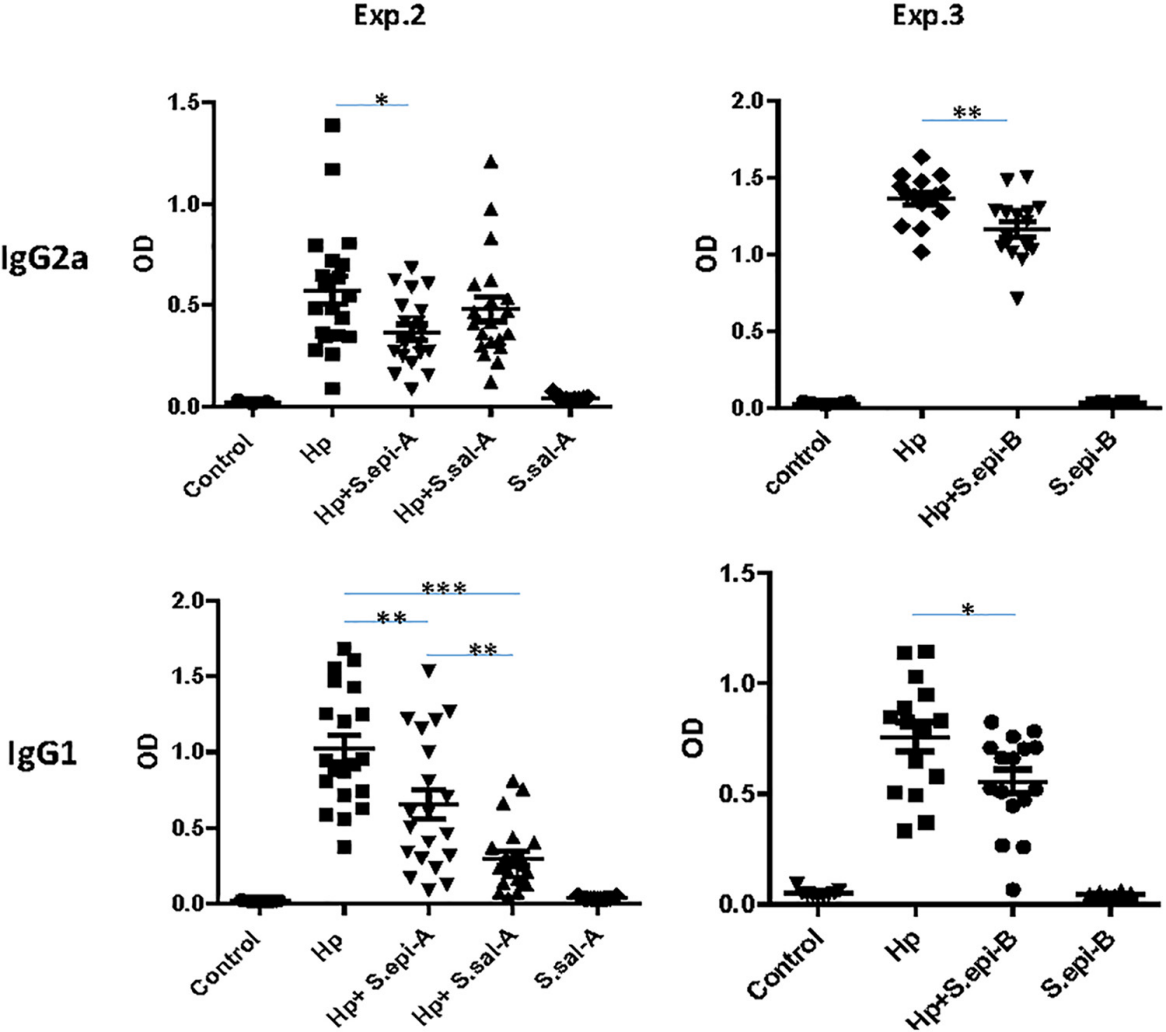
GF INS-GAS mouse serum antibody against H. pylori and S. epidermidis reduced proinflammatory anti-H. pylori isotype IgG2a. In experiments 1 and 2, *S. salivarius* and S. epidermidis both reduced anti-inflammatory anti-H. pylori isotype IgG1. Mice infected with H. pylori and *S. salivarius* developed the lowest anti-inflammatory IgG1 responses to H. pylori. *, *P* < 0.05; ** *P* < 0.01; ***, *P* < 0.001.

The observation of reduced gastric proinflammatory cytokines in mice coinfected with H. pylori and S. epidermidis compared to H. pylori alone was supported by reduced proinflammatory IgG2a responses to H. pylori in H. pylori-S. epidermidis-infected mice compared to mice infected with H. pylori alone (*P* < 0.05 in experiment 2 and *P* < 0.01 in experiment 3) (Fig. 5). In contrast, mice coinfected with H. pylori and *S. salivarius* developed the lowest anti-inflammatory IgG1 response to H. pylori (*P* < 0.001). The IgG1 response to H. pylori in mice coinfected with H. pylori and S. epidermidis was lower than that in mice infected with H. pylori alone (*P* < 0.01 in experiment 2 and *P* < 0.05 in experiment 3) but was greater than the response to H. pylori and *S. salivarius* (*P* < 0.01) (Fig. 5, experiment 2).

### (vi) Mice coinfected with H. pylori and *S. salivarius* had significantly higher numbers of FoxP3- and Ki67-positive cells in gastric tissues.

**FIG 6:**
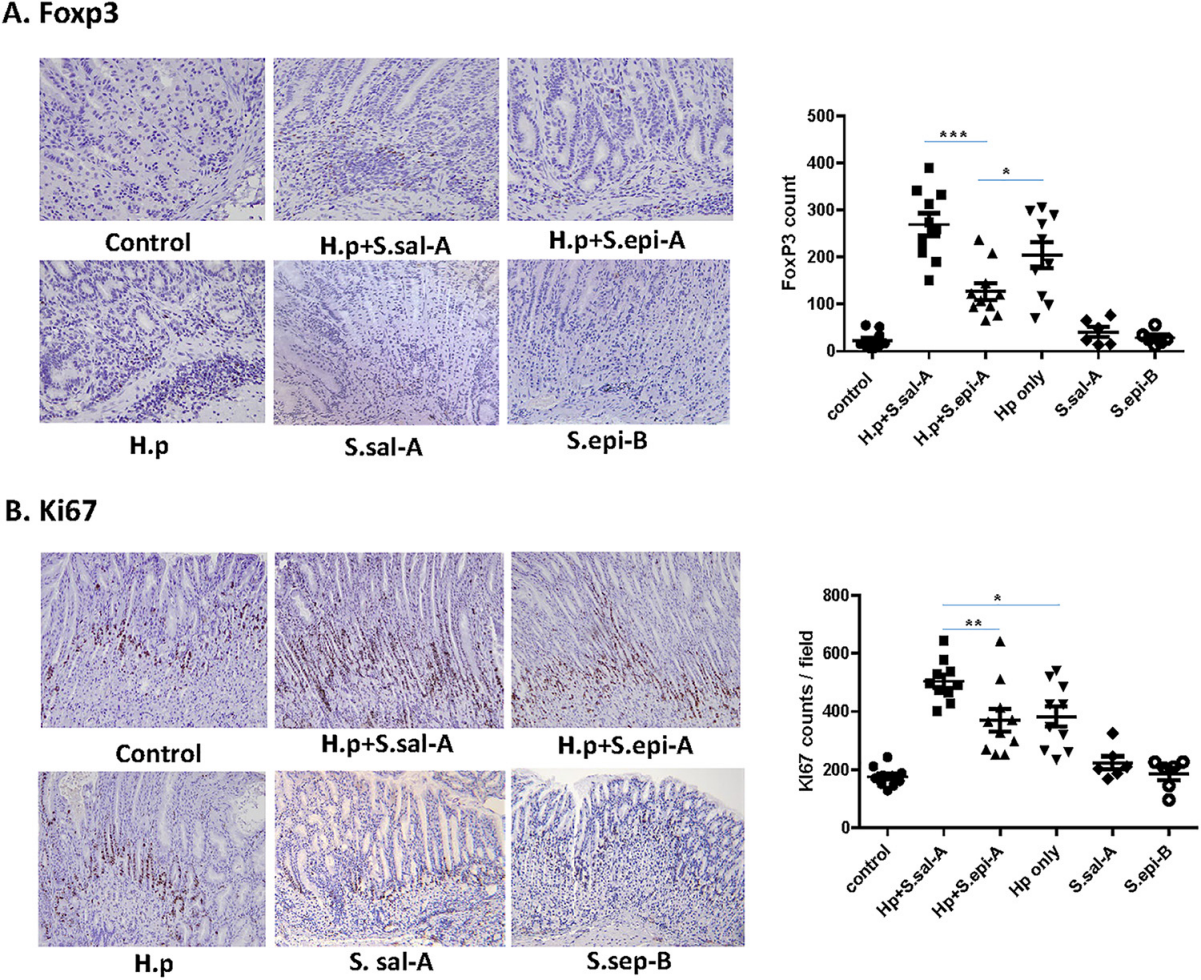
Quantifications of FoxP3 for Tregs (A) and the cell proliferation marker Ki67 (B) in the gastric tissue of INS-GAS mice were performed by IHC staining. The numbers of FoxP3- and Ki67-positive cells in the gastric mucosa and submucosa were counted for 10 fields (magnification, ×200) of mouse stomach corpus. *, *P* < 0.05; **, *P* < 0.01; ***, *P* < 0.001.

Immunohistochemistry (IHC) staining for FoxP3 and Ki67 was performed on paraffin-embedded gastric tissues. Increased numbers of FoxP3^+^ cells were noticed in the mice coinfected with H. pylori and *S. salivarius*. Mice coinfected with H. pylori and S. epidermidis had significantly lower numbers of FoxP3^+^ cells in their gastric mucosae than mice monocolonized with H. pylori (*P* < 0.05), as well as mice cocolonized with H. pylori and *S. salivarius* (*P* < 0.001) (Fig. 6A). Monoassociated S. epidermidis or *S. salivarius* mice had same levels of FoxP3^+^ cells as control mice (Fig. 6A).

Proliferating epithelial cells were detected by Ki67 IHC staining. In control mice, Ki67-positive cells were observed in the isthmus regions of the corpus mucosa. In all mice infected with H. pylori, the Ki67 staining was significantly higher (*P* < 0.05) than in the control mice. Proliferating cells in the corpus expanded from the isthmus regions to hypertrophic foveolar regions. Mice coinfected with H. pylori and *S. salivarius* had significantly higher Ki67 staining in the corpus than H. pylori-monoinfected mice (*P* < 0.05) and mice coinfected with H. pylori and S. epidermidis (*P* < 0.01). There was no difference in Ki67 staining between H. pylori-monoinfected mice and mice coinfected with H. pylori and S. epidermidis. S. epidermidis- and *S. salivarius*-monoinfected mice had the same levels of Ki67-positive cells as control mice (Fig. 6B).

## DISCUSSION

The human gastric microbiota composition and its changes after H. pylori colonization have been studied to investigate the mechanisms of H. pylori-induced gastric diseases (2). Several studies in the recent literature have demonstrated by culture methods that multiple species of bacteria were present in stomachs colonized with H. pylori, as well as stomachs not colonized with H. pylori. Hu et al. cultured 103 biopsy samples from H. pylori-positive patients from China and reported that 65% of patients had non-H. pylori bacteria, of which the dominant genera were Streptococcus, *Neisseria*, *Rothia*, and Staphylococcus (9). Khosravi et al. reported the culturable gastric microbiota of 215 Malaysian patients, including both 131 H. pylori-positive and 84 H. pylori-negative patients. They found that the most abundant bacteria at the genus level were Streptococcus, *Neisseria*, Klebsiella, and *Lactobacillus.* In their study, H. pylori colonization did not significantly affect the diversity of the gastric microbiota. Interestingly, Streptococcus species were associated with peptic ulcer disease (10). Another study cultured the gastric mucosa from 50 chronic gastritis and 53 clinically normal patients and found that the abundance of Streptococcus and *Neisseria* were markedly higher in the gastritis group than those in the normal group, suggesting that certain bacterial species may play a role in the development of gastritis rather than being only transient microbes (11). Stomach samples from 346 children in China revealed that the most abundant and culturable bacteria were Streptococcus, *Neisseria*, *Rothia*, and Staphylococcus (12). In another report, stomach samples from healthy volunteers in Brazil were surveyed for culturable bacteria; *Veillonella* sp., *Lactobacillus* sp., and *Clostridium* sp. were the dominant species (13). Like other studies, the most abundant species in our study were bacteria known to colonize the oral cavity or upper respiratory tract, such as Streptococcus, *Veillonella*, and Staphylococcus. The prevalence of *Bacillus* isolation was much higher than in other studies (Table S1). The aerobic bacterium *B. pumilus* naturally resides in soils and colonizes the roots of plants. Its abundance in LGCR regions may be related to their geographic location and the dietary habits of LGCR region inhabitants (14). A significantly higher prevalence of Staphylococcus spp. in the LGCR region was noted in our study. Our results in H. pylori-infected INS-GAS mice coinfected with S. epidermidis suggested that this bacterium may have a protective immunomodulatory effect on the progression of gastritis. The biological function of this organism involved in H. pylori-associated gastric cancer requires further investigation.

INS-GAS mice exhibit overexpression of human gastrin under the regulation of a rat insulin promoter. These mice have an age-dependent increase of parietal cell loss and gastric pathology. We previously reported that the normal intestinal and oral flora (IOF) in INS-GAS mice accelerated and promoted the progression of gastrointestinal intraepithelial neoplasia (GIN) (15), while antimicrobial treatment delayed onset of GIN in H. pylori*-*infected mice and, importantly, had the same effect in uninfected INS-GAS mice (16). Notably, H. pylori-free INS-GAS mice colonized with IOF developed GIN more quickly than GF INS-GAS mice, which remained free of GIN through 11 months of age (15). Similar results were observed in the *K19-Wnt1/C2mE* (Gan) mouse model of gastric adenocarcinoma (GAC). Specific-pathogen-free (SPF) Gan mice developed large gastric tumors, but gastric tumorigenesis was suppressed in GF Gan mice (17). These data suggest that non-H. pylori bacteria, including those considered potentially pathogenic or commensal microorganisms, can colonize the stomach and represent an important component when assessing GAC risk, particularly in H. pylori*-*infected, susceptible individuals (3, 5).

Although evidence supports a role for normal IOF in GAC, it is unknown if specific species of bacteria are required to modulate GAC progression or if the mechanism reflects gastric colonization with diverse microflora secondary to H. pylori-mediated gastric atrophy. Using the INS-GAS H. pylori mouse model, we evaluated if a restricted microbiota limited to three species of altered Schaedler’s flora (rASF), including ASF356 *Clostridium* species, ASF361 Lactobacillus murinus and ASF519 *Bacteroides* species, was sufficient to contribute to GIN incidence after development of gastric atrophy secondary to H. pylori infection (18–20). We demonstrated that gastric colonization with three species of ASF was comparable to colonization with diverse gastrointestinal microbiota in promoting gastritis and *in situ* gastric carcinoma (20).

The literature cites the presence of non-H. pylori urease-positive bacteria as the cause of false positives in H. pylori urea breath tests, as ^13^C urea is hydrolyzed by urease of these commensal bacteria (21–23). In our study, bacteria from multiple genera tested positive for urease activities. Although their urease activity levels were much lower (<15 U) than those of H. pylori (262 U), these bacteria might have a selective advantage in colonizing the stomach due to their ability to produce NH_3,_ which buffers the gastric acidity. We therefore selected two urease-positive bacteria, *S. salivarius*, present in both HGCR and LGCR patients, and S. epidermidis, predominantly present in the stomachs of LGCR patients, for further investigation.

In the current study, S. epidermidis was more commonly isolated from LGCR patients. In the INS-GAS mouse model, S. epidermidis efficiently colonized the stomach of H. pylori*-*infected INS-GAS mice. Importantly, even though S. epidermidis present in the mouse stomach with H. pylori did not change the severity of pathology compared with that in H. pylori-monoinfected mice, attendant reductions in the level of RNA expression of proinflammatory cytokine genes *Il-1β*, *Il-17A*, and *Ifn-γ*, as well as lower levels of *Il-22* and IgG2a expression, were noted. It has been reported that S. epidermidis strains, which can form biofilms, elicited production of lower levels of the proinflammatory cytokine genes *IL-1b*, *IFNr*, and *IL-12*, and increased levels of anti-inflammatory cytokines such as interleukin 13 (IL-13) in a primary human monocyte-derived macrophages model (24). Cogen et al. demonstrated that S. epidermidis*-*derived δ-toxin interacts with host-derived antimicrobial peptides, present in the innate immune system, to reduce the survival of group A Streptococcus, a human pathogen (25).

There were no statistically significant differences in H. pylori colonization levels in the stomachs of INS-GAS mice coinfected with either S. epidermidis or *S. salivarius*. This may be the result of H. pylori and non-H. pylori bacteria being present in different colonization niches of the stomach. Brandi et al. have reported that non-H. pylori bacteria are grouped into clusters of different sizes localized mainly over the mucus layer of the stomach and only occasionally can be found embedded in the mucus and in direct contact with gastric microvilli in human biopsy samples (21). While H. pylori is spiral shaped with a bundle of unipolar flagella, it is highly motile and efficiently penetrates the mucus layers to reside in the internal mucus layer adjacent to epithelial cells (26, 27). H. pylori can also establish colonies deep in the gastric glands, and these populations can expand in size and colonize adjacent glands to provide protected niches during chronic infection (28).

*S. salivarius* is among the members of the healthy human oral microbiota which are regularly found in human stomach samples. It has been reported that an increased abundance of members of the genus Streptococcus can be found in antral gastritis and peptic ulcer diseases as well as in patients with multifocal atrophic gastritis with intestinal metaplasia (9, 10, 29). *S. salivarius* also persistently colonizes the stomachs of H. pylori-infected INS-GAS mice. Cocolonization by H. pylori and *S. salivarius* induced robust pathology and statistically higher pathology scores compared to H. pylori monoinfection of INS-GAS mice and coinfection with H. pylori and S. epidermidis. Tissue mRNA levels of the cytokines *Il-1β*, *Il-17A*, *Ifn-γ*, and *Tnf-α* and the antimicrobial peptides *Reg-3β* and *Reg-3γ*, as well as serum IgG2a, were comparable among the monoassociated H. pylori group and the H. pylori-*S. salivarius* group. Of interest, the expression levels of *Il-10* RNA in mice coinfected with *S. salivarius* and H. pylori were lower than in H. pylori-monoinfected mice. The presence of these chemokines during H. pylori infection leads to the recruitment of immune cells, including neutrophils, macrophages, dendritic cells (DCs), NK cells, and lymphocytes, to infection sites and causes persistent chronic inflammation and gastritis (30). In GF mice coinfected with *S. salivarius* and H. pylori, this non-H. pylori bacterium may serve as an additional proinflammatory stimulus augmenting the severity of H. pylori-induced pathology. H. pylori colonization levels were comparable between H. pylori-monoinfected mice and those coinfected with H. pylori and *S. salivarius*. The mechanism whereby *S. salivarius* increased pathology in H. pylori*-*infected mice is unknown. Certain strains of *S. salivarius* exhibit anti-inflammatory properties in the oral and digestive tract as well as affecting IL-8 secretion and innate immune responses in bronchial and pharyngeal epithelial cells (31–34). Nevertheless, studies involving the intrinsic immunomodulatory effect of *S. salivarius* on cells related to the immune system, including activated immune cells and overexpression of proinflammatory cytokines, have been reported. (35–37).

H. pylori infection induces gastric epithelial cells to produce the proinflammatory cytokines and chemokines such as *Il-8*, *Il-1β*, *Il-6*, *Il-17A*, *Il-22*, *Ifn-γ*, and *Tnf-α* to promote gastric inflammation by neutrophils and macrophages infiltrations and initiation of Th1 immune responses (38). Chronic gastritis can progress to gastric atrophy, intestinal metaplasia, and then dysplasia, leading to neoplastic transformation. Complex control of chronic inflammation in H. pylori infection is mediated by a balance of T_H_1/T_H_2/T_H_17 and regulatory-T-cell (Treg) responses (39). H. pylori infection induces Th1 and Th17 responses, which cause chronic inflammation, but is insufficient to clear H. pylori infection. Persistent infection results in the expansion of Tregs to reduce inflammatory response to protect against exaggerated tissue damage. The transcription factor forkhead box protein 3 (FoxP3) is the hallmark of regulatory T cells. It has been frequently reported that Tregs are increased in gastric cancer patients and patients with severe gastritis (40, 41). Gastric FoxP3^+^ cells were positively associated with the severity of H. pylori-induced gastric lesions in INS-GAS mice (42). FoxP3^+^ Tregs were significantly more abundant in the gastric epithelial cells of mice coinfected with *S. salivarius* and H. pylori in our study. IL-17 stimulates gastric epithelial cells to release IL-8, which recruits neutrophils and enhances chronic inflammation (43). IL-17A and IL-22 are also associated with antimicrobial responses and the control of bacterial colonization (44). In our GF INS-GAS mouse model, the most significantly altered cytokine genes in coinfected mice were *Il-1β*, *Il-17A*, and *Il-22*, which were significantly upregulated in mice coinfected with H. pylori and *S. salivarius* but significantly downregulated in mice coinfected with H. pylori and S. epidermidis. Mice coinfected with H. pylori and *S. salivarius* had significantly higher numbers of proliferating epithelial cells detected by Ki67 staining in the stomach than H. pylori-monoinfected mice (*P* < 0.05) and mice coinfected with H. pylori and S. epidermidis (*P* < 0.01).

This study confirms our earlier microbiome analysis of H. pylori*-*infected Colombian patients, demonstrating that both high- and low-gastric-cancer-risk patients have their stomachs colonized by a multitude of non-H. pylori bacteria (7). One of these bacteria, Staphylococcus sp. (OTU566), which had 99% sequence identity with S. epidermidis, was identified from the stomachs of LGCR patients at a much higher frequency than HGCR patients, which is consistent with our culture-based study on a similar patient population. Using a H. pylori-infected INS-GAS model, we demonstrated that S. epidermidis cocolonization with H. pylori attenuated proinflammatory responses. We attribute this finding to possible immunomodulating properties of S. epidermidis and its inhibitory effects of inflammation-associated H. pylori gastritis. Another possibility is that S. epidermidis also has antimicrobial effects on H. pylori and other commensal gastric colonizers, which could also afford protection in the gastric compartment of patients infected with H. pylori and other gastric compartment-colonizing microbes. It is of interest that in our study, *S. salivarius* and H. pylori induced statistically more severe gastritis than either H. pylori monoinfection or H. pylori-S. epidermidis coinfection. We hypothesize that the *S. salivarius* strain from the stomach of a Colombian patient elicited a stronger Th1- and Th17-mediated immune response, allowing the overexpression of proinflammatory cytokine genes *Il-1β*, *Il-17A*, and *Ifn-γ*, which promoted chronic gastric inflammation and more severe gastric hyperplasia and dysplasia in H. pylori-infected INS-GAS mice cocolonized with *S. salivarius*.

It is appreciated that GF mice do not recapitulate all facets of H. pylori disease in human. Unlike humans with an intact complex microbiota structure, the lack of microbes in germfree mice allows them to be challenged with defined populations of bacteria (i.e., H. pylori with S. epidermidis*/S. salivarius*) to evaluate how specific microbes affect disease states (i.e., gastric inflammation and cancer). In summary, this study reinforces the argument that non-H. pylori bacteria colonize the stomachs of humans and play a role in the severity of H. pylori-induced gastric cancer. Further, using GF INS-GAS mice, it was demonstrated that the urease-positive bacteria S. epidermidis and *S. salivarius* isolated from Colombian patients have the ability to promote or attenuate H. pylori associated gastritis.

## MATERIALS AND METHODS

### Study population, samples, and histopathology.

As previously cited, subjects between 40 and 60 years of age with dyspeptic symptoms that warranted upper gastrointestinal tract endoscopy were recruited in Tumaco (LGCR) and Túquerres (HGCR) in 2010. Subjects that had received proton pump inhibitors, H2 receptor antagonists, or antimicrobials during the 30-day period previous to the endoscopic procedure were excluded from this study (7). Other exclusion criteria were major diseases or previous gastrectomy. Participation was voluntary, and informed consent was obtained from all participants. The ethics committees of the participating hospitals in Nariño and the Universidad del Valle in Cali, Colombia, and the Institutional Review Board of Vanderbilt University approved all study protocols, and all experiments were performed in accordance with the relevant guidelines and regulations. A sample population of 37 patients (19 from the HGCR region and 18 from the LGCR region) consisting of 17 males (average age, 50 years) and 20 females (average age, 49 years) were recruited for gastric endoscopy in this study. By histologic diagnosis, 32 patients had non-atrophic gastritis (NAG), 2 had multifocal atrophic gastritis (MAG), and 3 had MAG with intestinal metaplasia. Twenty of the patients were H. pylori positive by Warthin-Starry stain.

### Bacterial culture methods for gastric biopsy specimens from patients.

Antral biopsy specimens were frozen at −80°C in brain heart infusion (BHI) broth containing 20% glycerol. The biopsy specimens were thawed in an anaerobic atmosphere (10% CO_2_, 10% H_2_, 80% N_2_) and were homogenized in BHI with 20% glycerol with tissue grinders. The homogenate was divided into aliquots to isolate bacteria under diverse culture conditions. For aerobic culture, the homogenates were plated onto chocolate agar, blood agar, MacConkey agar, and Brucella broth medium containing 10% fetal calf serum (FCS). The plates were incubated at 37°C in 5% CO_2_ for 24 to 48 h. For anaerobic culture, the homogenates were plated onto prereduced Brucella blood agar plates (BBL) and inoculated into thioglycolate broth. The cultures were incubated at 37°C in an anaerobic chamber (Coy Lab Products) with mixed gas (10% CO_2_, 10% H_2_, 80% N_2_) for 48 h. For microaerobic culture to detect the growth of H. pylori, the homogenates were plated onto H. pylori selective plates (45) and Brucella blood agar plates after passing through a 0.65-μm syringe filter. The plates were placed into a vented jar filled with mixed gas (10% CO_2_, 10% H_2_, 80% N_2_) and incubated at 37°C for up to 3 weeks. The plates were checked every 2 to 3 days for growth. All bacterial strains isolated from the different culture conditions were identified by 16S rRNA sequencing.

### Urease activity assay.

All bacterial isolates showing a distinct urease-positive reaction on urea agar slants were further evaluated for their urease activity. Briefly, the bacterial cells were grown in Trypticase soy broth (TSB) for 24 h with shaking at 150 rpm at 37°C. The cultures were washed and resuspended in phosphate-buffered saline (PBS) and adjusted to a 0.5 MacFarland turbidity standard. Urease activity of the bacterial isolates was measured using a urease activity assay kit (MAK120; Sigma-Aldrich) following the manufacturer’s instructions. Urease activity was measured in duplicate or triplicate for each strain on separate occasions. One unit of urease is the amount of enzyme that catalyzes the formation of 1.0 μmol of ammonia per min at pH 7.0.

### Germ-free-mouse experiments.

The animal protocol was approved by the Massachusetts Institute of Technology Committee of Animal Care. GF INS-GAS mice on an FVB/N background [Tg(Ins1-GAS) 1Sbr] were maintained in a facility accredited by AAALAC International. GF mice were housed in sterile isolators on autoclaved hardwood bedding in solid-bottomed polycarbonate cages and fed autoclaved rodent diet (Prolab RMH 3000; PMI Nutrition International), and sterile water was provided *ad libitum*. Isolators for GF control mice were surveyed every other week and confirmed negative for microbial contaminants by culture, PCR using eubacterial primers, and Gram-stained fecal smears.

Seven- to 8-week-old male and female INS-GAS mice were infected by oral gavage with 200 μL of an H. pylori (SS1 strain) suspension at 1 unit of optical density at 600 nm (OD_600_)/mL (∼2 × 10^8^ CFU) on alternate days for a total of 3 doses (46). Coinfected mice were orally infected with H. pylori followed in 1 week by further dosing 200 μL of 1 OD_600_/mL (∼2 × 10^8^ CFU) of either *S. salivarius*, S. epidermidis, or both organisms (Table 2). Mice were necropsied at 5 months postinfection.

### (i) Necropsy.

Immediately following CO_2_ euthanasia of the mice, blood was collected via cardiac puncture; serum was separated and stored at −80°C. The stomach and proximal duodenum were aseptically removed and incised along the greater curvature. Four individual linear gastric strips from the lesser curvature were sectioned for culture, flash frozen for RNA analysis, and stored at −80°C for DNA extraction or preserved in 10% neutral buffered formalin for histopathologic evaluation (47). Gastric lesions were graded by a comparative pathologist who was blind to sample identity on an ascending scale from 0 to 4 for inflammation, epithelial defects, atrophy, hyperplasia, pseudopyloric metaplasia, dysplasia, and mucous metaplasia. A gastric histologic activity index (GHAI) was generated by combining scores for all criteria.

### (ii) Quantification of H. pylori.

A correlation assay between copy number in quantitative PCR and CFU in bacterial culture was performed. H. pylori SS1 strain was grown microaerobically on 5% sheep blood agar plates for 24 h. The bacterial suspension was adjusted to 1 OD_600_/mL and serially diluted (10×) in Brucella broth. Bacterial suspensions were subjected in parallel to DNA extraction for qPCR and microanaerobic culture for bacterial CFU.

Colonization levels of gastric H. pylori SS1 were quantified using qPCR in the 7500 FAST real-time PCR system (Thermo Fisher Scientific, Waltham, MA) as previously described (48). Copy numbers of H. pylori were normalized to micrograms of mouse chromosomal DNA in the samples measured by qPCR using the 18S rRNA gene-based primers and probe mixture (Thermo Fisher Scientific, Waltham, MA).

### (iii) CFU counts for *S. salivarius* or S. epidermidis in mouse stomach samples.

Mouse stomach samples were weighed and homogenized with 1 mL of Brucella broth using a glass tissue grinder. The homogenate was diluted 10- and 100-fold in Brucella broth. Fifty microliters of each dilution was plated on CPS Elite plates (bioMérieux, Cambridge, MA) and incubated in a 5% CO_2_ incubator at 37°C for 24 h. Blue colonies indicated growth of *S. salivarius*; white colonies indicated growth of S. epidermidis.

### (iv) Cytokine mRNA expression profiles in stomach samples of GF INS-GAS mice.

RNA was extracted from stomach tissue samples using the TRIzol reagent (Thermo Fisher Scientific, Waltham, MA). Total RNA (2 μg) was converted to cDNA using a high-capacity cDNA Archive kit following the manufacturer protocol (Thermo Fisher Scientific, Waltham, MA). cDNA levels for *Tnf-α*, *Ifn-γ*, *Il-1β*, *Il-22*, *Il-10*, *Il-17A*, and *iNos* mRNA were measured by quantitative PCR using commercial primers and probes for each cytokine. Briefly, duplicate 20-μL reaction mixtures contained 5 μL of cDNA, 1 μL of a commercial 20× primer-probe solution, 10 μL of 2× master mix (Thermo Fisher Scientific, Waltham, MA), and 4 μL of double-distilled H_2_O. Relative expression of mRNA from infected and control samples was calculated using the comparative cycle threshold (*C_T_*) method with RNA input standardized between samples by expression levels of the endogenous reference gene, *Gapdh*. Results from duplicate samples were plotted as fold changes between cells or tissues from infected and uninfected controls.

### (v) Serology.

Serum Th1-associated IgG2a and Th2-associated IgG1 responses to sonicated antigens of H. pylori, S. epidermidis and *S. salivarius* were measured by enzyme-linked immunosorbent assay (ELISA). To prepare antigens, H. pylori SS1 was grown on blood agar plates under microaerobic condition for 48 h, *S. salivarius* (MIT14-1770 C1) was grown in M17 medium, and S. epidermidis (MIT 14-1777 C6; MIT 14-1779 M5) was grown on Brucella broth overnight. Bacterial cells were pelleted and washed once with PBS by centrifugation at 12,000 rpm for 5 min. Pellets were resuspended in 5 mL of PBS and then sonicated on ice with an Ultrasonic liquid processor (Misonix, Newtown, CT) using the following program: amplitude, 35; power, 7 W at 30-s intervals for a total of 5 min with 1-min breaks between intervals. Sonicated samples were centrifuged at 12,000 rpm for 10 min at 4°C and filtered through a 0.2-μm syringe filter to remove cell debris.

For serum IgG measurement, 96-well Immulon II plates (Thermo Fischer Scientific, Waltham, MA) were coated with antigen at a concentration of 10 μg/mL and incubated overnight at 4°C. Wells were blocked with 200 μL per well of 2% bovine serum albumin (BSA) in PBS. Serum samples were diluted 1:100 in PBS supplemented with 1% FBS and plated at 100 μL/well. Biotinylated monoclonal anti-mouse antibodies produced by clones A85-1 (specific for mouse IgG1) and 5.7 (specific for mouse IgG2; BD Biosciences, San Jose, CA) were diluted 1:2,000 and used as secondary antibodies (100 μL/well). Incubation with ExtrAvidin-peroxidase (1:2,000, 100 μL/well) (Sigma, St. Louis, MO) was followed by 2,2′-azinobis (3-ethylbenzthiazoline-6-sulfonic acid) diammonium salt (ABTS) substrate (Kirkegaard and Perry Laboratories, Gaithersburg, MD) for color development at 405/590 nm.

### (vi) Immunohistochemistry for Foxp3^±^ and Ki67 cells.

Foxp3 immunochemistry to detect regulatory T cells (Treg) was performed with Foxp3 antibody (1:50; FJK-16S; eBioscience, San Diego, CA). For assessment of epithelial cell proliferation, Ki67 (1:50, BD Biosciences, San Jose, CA) labeling was performed as described previously (49). Paraffin-embedded gastric tissues from 10 mice of each group were selected for these assays. The numbers of cells expressing nuclear staining for Foxp3 and for Ki67 were counted on 10 fields (×200) per stomach and are reported as the average number of positive cells per field.

### Statistical analysis.

Fisher’s exact test was used for bacteria species isolated from human biopsy samples. Shannon index (H) was used to analyze bacterial diversities in different populations and was computed as follows:

$$H=-\overset{s}{\underset{i=i}{\sum }}p_{i}lnp_{i}$$

with the variance computed as

$$s_{H}^{2}=\frac{\sum p{\left(\mathrm{lnp}\right)}^{2}\;-\;{\left(\sum p\mathrm{lnp}\right)}^{2}}{N}\;+\;\frac{S\;-\;1}{2N^{2}}$$

where *p* is the proportion of a species toward the total, *S* is the number of species, and *N* is the total abundance. The Hutcheson *t* test was used to compare the diversity between communities of interest (50).

GF mouse stomach lesion scores were analyzed using the Mann-Whitney U nonparametric test for ordinal data; H. pylori colonization, levels of cytokine mRNA expression, and serology results evaluated were compared by Student's *t* test. Statistical analysis was performed using R 3.6.3 (51) and GraphPad Prism 5.0 (GraphPad Software, Inc., La Jolla, CA). Results were considered significant at a *P* value of <0.05.
