# Supplementary material for: Gastric Non-Helicobacter pylori Urease-Positive Staphylococcus epidermidis and Streptococcus salivarius Isolated from Humans Have Contrasting Effects on H. pylori-Associated Gastric Pathology and Host Immune Responses in a Murine Model of Gastric Cancer
Source: mSphere. 2022 Feb 9;7(1):e00772-21. doi: 10.1128/msphere.00772-21 (PMC8826947; doi:10.1128/msphere.00772-21)
Supplement: TABLE S1 [file msphere.00772-21-st001.docx]

|  |  |  |  |  |  |  | **HGCR** | | **LGCR** | |
| --- | --- | --- | --- | --- | --- | --- | --- | --- | --- | --- |
| **phylum** | **Class** | **order** | **family** | **Genus** | **n*** | **species** | **HP+(n/10)** | **HP-(n/9)** | **HP+(n/10)** | **HP-(n/8)** |
| **Actinobacteria** | **Actinobacteria** | **Actinomycetales** | ***Actinomycetaceae*** | ***Actinomyces*** | **4** |  | **4** |  |  |  |
|  |  |  |  |  |  | ***Actinomyces odontolyticus*** | **3** |  |  |  |
|  |  |  |  |  |  | ***Actinomyces oris*** | **1** |  |  |  |
| **Actinobacteria** | **Actinobacteria** | **Actinomycetales** | ***Gordoniaceae*** | ***Gordonia*** | **2** |  |  | **1** | **1** |  |
|  |  |  |  |  |  | ***Gordonia alkanivorans*** |  | **1** |  |  |
|  |  |  |  |  |  | ***Gordonia polyisoprenivorans*** |  |  | **1** |  |
| **Actinobacteria** | **Actinobacteria** | **Actinomycetales** | ***Streptomycetaceae*** | ***Streptomyces*** | **2** |  | **2** |  |  |  |
|  |  |  |  |  |  | ***Streptomyces collinus*** | **1** |  |  |  |
|  |  |  |  |  |  | ***Streptomyces diastaticus*** | **1** |  |  |  |
| **Actinobacteria** | **Actinobacteria** | **Micrococcales** | ***Micrococcaceae*** | ***Arthrobacter*** | **1** |  | **1** |  |  |  |
|  |  |  |  |  |  | ***Arthrobacter oxydans*** | **1** |  |  |  |
| **Actinobacteria** | **Actinobacteria** | **Micrococcales** | ***Micrococcaceae*** | ***Rothia*** | **2** |  | **2** |  |  |  |
|  |  |  |  |  |  | ***Rothia mucilaginosa*** | **1** |  |  |  |
|  |  |  |  |  |  | ***Rothia sp. oral taxon*** | **1** |  |  |  |
| **Actinobacteria** | **Actinobacteria** | **Propionibacteriales** | ***Propionibacteriaceae*** | ***Propionibacterium*** | **2** |  |  | **1** | **1** |  |
|  |  |  |  |  |  | ***Propionibacterium acnes*** |  | **1** | **1** |  |
| **Actinobacteria** | **Coriobacteriia** | **Coriobacteriales** | ***Atopobiaceae*** | ***Atopobium*** | **3** |  | **2** | **1** |  |  |
|  |  |  |  |  |  | ***Atopobium parvulum*** | **1** | **1** |  |  |
|  |  |  |  |  |  | ***Atopobium rimae*** | **1** |  |  |  |
| **Bacteroidetes** | **Bacteroidia** | **Bacteroidales** | ***Prevotellaceae*** | ***Prevotella*** | **2** |  | **1** |  | **1** |  |
|  |  |  |  |  |  | ***Prevotella melaninogenica*** | **1** |  | **1** |  |
| **Firmicutes** | **Bacilli** | **Bacillales** | ***Bacillaceae*** | ***Bacillus*** | **18** |  | **3** | **4** | **6** | **5** |
|  |  |  |  |  |  | ***Bacillus altitudinis*** |  |  | **1** |  |
|  |  |  |  |  |  | ***Bacillus amlyloliquefaciens*** | **1** |  |  |  |
|  |  |  |  |  |  | ***Bacillus megaterium*** |  |  | **1** | **1** |
|  |  |  |  |  |  | ***Bacillus pumilus*** |  | **1** | **4** | **3** |
|  |  |  |  |  |  | ***Bacillus subtilis*** | **1** | **2** | **1** | **2** |
|  |  |  |  |  |  | ***Bacillus thuringensis*** | **1** | **2** |  |  |
| **Firmicutes** | **Bacilli** | **Bacillales** | ***Bacillales Family XI.*** | ***Gemella*** | **1** |  | **1** |  |  |  |
|  |  |  |  |  |  | ***Gemella sanguinis*** | **1** |  |  |  |
| **Firmicutes** | **Bacilli** | **Bacillales** | [***Paenibacillaceae***](https://www.google.com/search?client=safari&rls=en&q=Paenibacillaceae&stick=H4sIAAAAAAAAAOPgE-LUz9U3MCssMzRQAjMti7LiDbUss5Ot9JMy83Py0yv184vSE_Myi3Pjk3MSi4sz0zKTE0sy8_OsMjLTM1KLFFBFF7EKBCSm5mUmJSZn5uQkJqcmpu5gZQQAA0z_N2kAAAA&sa=X&ved=2ahUKEwjluMz73LPpAhWDhXIEHcqnDrQQmxMoATAgegQICxAD) | ***Paenibacillus*** | **1** |  |  | **1** |  |  |
|  |  |  |  |  |  | ***Paenibacillus odorifer*** |  | **1** |  |  |
| **Firmicutes** | **Bacilli** | **Bacillales** | ***Staphylococcaceae*** | ***Staphylococcus*** | **14** |  | **2** | **2** | **4** | **6** |
|  |  |  |  |  |  | ***Staphylococcus pasteuri or warneri*** | **1** |  | **1** |  |
|  |  |  |  |  |  | ***Staphylococcus capitis*** |  |  | **1** |  |
|  |  |  |  |  |  | ***Staphylococcus caprae*** |  |  | **1** |  |
|  |  |  |  |  |  | ***Staphylococcus condimenti*** |  | **1** | **1** |  |
|  |  |  |  |  |  | ***Staphylococcus epidermidis*** | **2** | **1** | **3** | **6** |
| **Firmicutes** | **Bacilli** | **Lactobacillales** | ***Aerococcusceae*** | ***Aerococcus*** | **1** |  |  | **1** |  |  |
|  |  |  |  |  |  | ***Aerococcus viridans*** |  | **1** |  |  |
| **Firmicutes** | **Bacilli** | **Lactobacillales** | ***Lactobacillaceae*** | ***Lactobacillus*** | **2** |  | **1** |  |  | **1** |
|  |  |  |  |  |  | ***Lactobacillus crispatus*** | **1** |  |  |  |
|  |  |  |  |  |  | ***Lactobacillus gasseri*** | **1** |  |  | **1** |
|  |  |  |  |  |  | ***Lactobacillus oris*** | **1** |  |  |  |
|  |  |  |  |  |  | ***Lactobacillus vaginalis*** |  |  |  | **1** |
| **Firmicutes** | **Bacilli** | **Lactobacillales** | ***Streptococcaceae*** | ***Enterococcus*** | **1** |  |  |  |  | **1** |
|  |  |  |  |  |  | ***Enterococcus faecalis*** |  |  |  | **1** |
| **Firmicutes** | **Bacilli** | **Lactobacillales** | ***Streptococcaceae*** | ***Streptococcus*** | **17** |  | **6** | **5** | **2** | **4** |
|  |  |  |  |  |  | ***Streptococcus anginosus*** | **1** | **1** |  |  |
|  |  |  |  |  |  | ***Streptococcus australis*** | **1** |  | **1** | **1** |
|  |  |  |  |  |  | ***Streptococcus constellatus*** | **2** | **1** |  |  |
|  |  |  |  |  |  | ***Streptococcus cristatus*** | **2** |  |  |  |
|  |  |  |  |  |  | ***Streptococcus gordonii*** |  |  | **1** | **1** |
|  |  |  |  |  |  | ***Streptococcus infantis*** |  | **1** |  |  |
|  |  |  |  |  |  | ***Streptococcus mitis*** | **3** |  | **1** | **3** |
|  |  |  |  |  |  | ***Streptococcus oralis*** | **2** |  |  | **1** |
|  |  |  |  |  |  | ***Streptococcus parasanguinis*** | **2** | **2** | **1** | **3** |
|  |  |  |  |  |  | ***Streptococcus salivarius*** | **3** | **2** | **2** | **3** |
|  |  |  |  |  |  | ***Streptococcus sanguinis*** |  |  | **1** | **1** |
|  |  |  |  |  |  | ***Streptococcus sp (oral strain)*** | **1** |  |  | **1** |
| **Firmicutes** | **Clostridia** | **Clostridiales** | ***Clostridiales Family XIII.*** | ***Mogibacterium*** | **1** |  | **1** |  |  |  |
|  |  |  |  |  |  | ***Mogibacterium diversum*** | **1** |  |  |  |
| **Firmicutes** | [**Erysipelotrichia**](https://en.wikipedia.org/wiki/Erysipelotrichia) | [**Erysipelotrichales**](https://en.wikipedia.org/wiki/Erysipelotrichales) | [***Erysipelotrichidae***](https://en.wikipedia.org/wiki/Erysipelotrichidae) | ***Solobacterium*** | **1** |  | **1** |  |  |  |
|  |  |  |  |  |  | ***Solobacterium moorei*** | **1** |  |  |  |
| **Firmicutes** | **Negativicutes** | **Veillonellales** | ***Veillonellaceae*** | ***Veillonella*** | **7** |  | **4** |  | **1** | **2** |
|  |  |  |  |  |  | ***Veillonella atypica*** | **2** |  | **1** |  |
|  |  |  |  |  |  | ***Veillonella prarvula*** | **2** |  |  |  |
|  |  |  |  |  |  | ***Veillonella rogosae*** |  |  |  | **1** |
|  |  |  |  |  |  | ***Veillonella despar*** |  |  |  | **1** |
| **Proteobacteria** | **Betaproteobacteria** | **Neisseriales** | ***Neisseriaceae*** | ***Neisseria*** | **2** |  | **1** |  | **1** |  |
|  |  |  |  |  |  | ***Neisseria mucosa*** | **1** |  | **1** |  |
|  |  |  |  |  |  | ***Neisseria subflava*** |  |  | **1** |  |
| **Proteobacteria** | **Gammaproteobacteria** | **Enterobacterales** | ***Enterobacteriaceae*** | ***Citrobacter*** | **1** |  |  | **1** |  |  |
|  |  |  |  |  |  | ***Citrobacter freundii*** |  | **1** |  |  |
| **Proteobacteria** | **Gammaproteobacteria** | **Enterobacterales** | ***Enterobacteriaceae*** | ***Escherichia*** | **1** |  |  | **1** |  |  |
|  |  |  |  |  |  | ***Escherichia coli*** |  | **1** |  |  |
| **Proteobacteria** | **Gammaproteobacteria** | **Pseudomonadales** | ***Moraxellaceae*** | ***Moraxella*** | **1** |  | **1** |  |  |  |
|  |  |  |  |  |  | ***Moraxella osloensis*** | **1** |  |  |  |
| **Proteobacteria** | **Gammaproteobacteria** | [**Xanthomonadales**](https://en.wikipedia.org/wiki/Xanthomonadales) | [***Xanthomonadaceae***](https://en.wikipedia.org/wiki/Xanthomonadaceae) | ***Xanthyomonas*** | **1** |  |  | **1** |  |  |
|  |  |  |  |  |  | ***Xanthyomonas sp.*** |  | **1** |  |  |
| **Proteobacteria** | **Epsilonproteobacteria** | **Campylobacterales** | ***Campylobacteraceae*** | ***Campylobacter*** | **1** |  | **1** |  |  |  |
|  |  |  |  |  |  | ***Campylobacter jejuni*** | **1** |  |  |  |
| **Proteobacteria** | **Epsilonproteobacteria** | **Campylobacterales** | ***Helicobacteraceae*** | ***Helicobacter*** | **17** |  | **7** |  | **10** |  |
|  |  |  |  |  |  | ***Helicobacter pylori*** | **7** |  | **10** |  |

**Table S1. Prevalence of bacteria isolated from stomach biopsy samples of HGCR and LGCR populations**.
