# Supplementary figures and images for: Gastric Non-Helicobacter pylori Urease-Positive Staphylococcus epidermidis and Streptococcus salivarius Isolated from Humans Have Contrasting Effects on H. pylori-Associated Gastric Pathology and Host Immune Responses in a Murine Model of Gastric Cancer
Source: mSphere. 2022 Feb 9;7(1):e00772-21. doi: 10.1128/msphere.00772-21 (PMC8826947; doi:10.1128/msphere.00772-21)

Supplemental Figure S1.

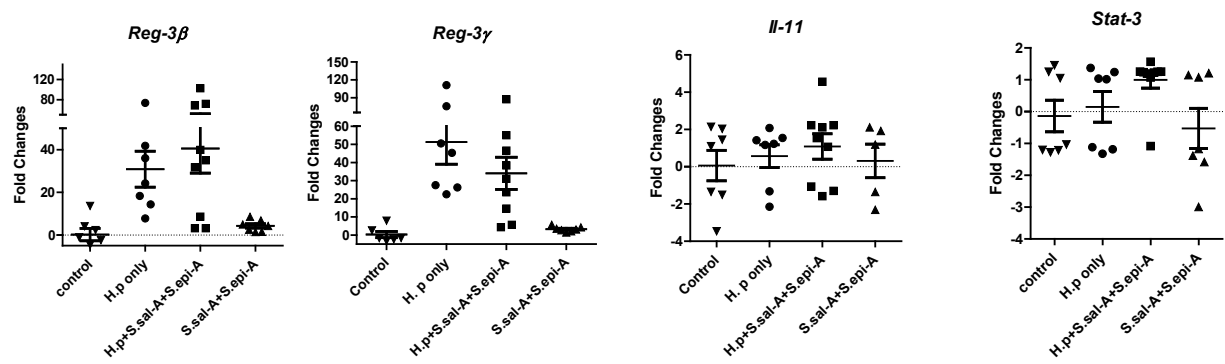

Supplement: FIG S1 [file msphere.00772-21-sf001.pdf]

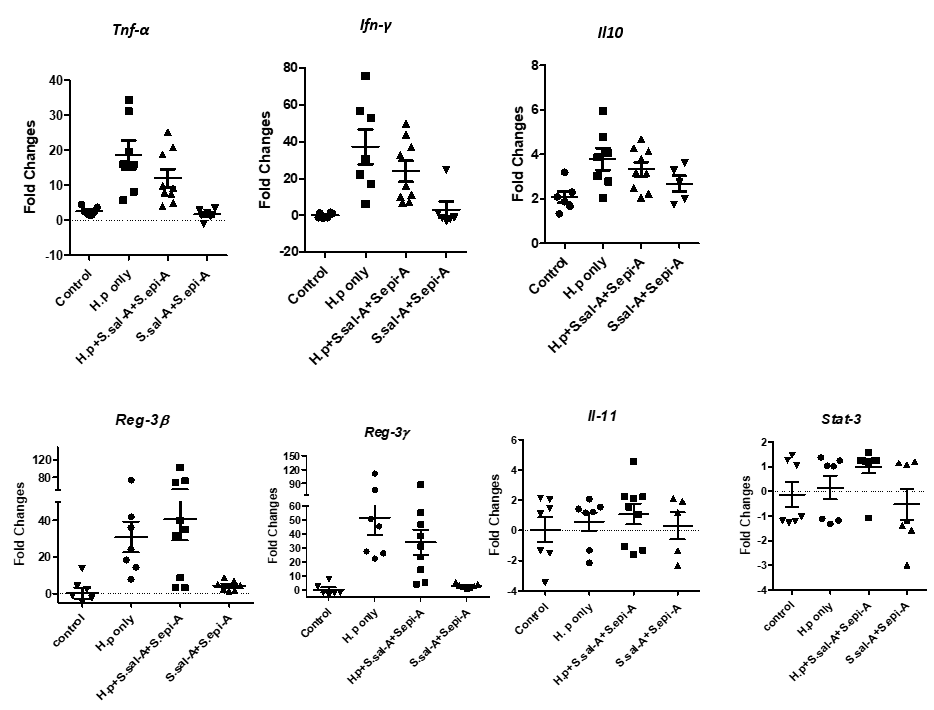

Supplement: FIG S2 [file msphere.00772-21-sf002.tif]
